# Decision-Making Time and Neuromuscular Coordination in Youth and Senior Soccer Goalkeepers

**DOI:** 10.3390/s23094483

**Published:** 2023-05-04

**Authors:** Katarzyna Piechota, Edyta Majorczyk

**Affiliations:** Faculty of Physical Education and Physiotherapy, Opole University of Technology, 45-758 Opole, Poland; e.majorczyk@po.edu.pl

**Keywords:** perception, focus, movement pattern, timing of muscle activation, soccer goalkeeper, youth and senior groups

## Abstract

The aim of this study was to compare soccer goalkeepers’ decision-making times following a shot on goal and to determine goalkeepers’ movement pattern structures using EMG in a typical game situation (two-on-one). Two groups of goalkeepers (*n* = 60) took part in the study: Group A, the senior group (22.00 ± 2.35 years of age), and Group B, the youth group (15.38 ± 1.32 years of age). The goalkeepers’ decision-making times were measured by using EMG from the moment the attacker struck the ball until the completion of the saving action by the goalkeeper. Subsequently, the goalkeepers’ movement pattern structure was determined (for both Groups A and B), and the values of muscle bioelectrical tension during a typical defensive situation in training conditions were revealed. The findings clearly indicate a significantly (*p* = 0.001) shorter decision-making time in experienced goalkeepers (250–260 ms) than in novices (300–320 ms). In addition, the movement pattern structure confirmed the hypotheses on the economization of effort and the visual-muscular coordination of the postural muscles (calf muscles) that affect soccer goalkeepers. The study also demonstrated a lower bioelectric tension of the gastrocnemius muscle (GAS.MED. RT—*p* = 0.008; GAS.LAT. RT—*p* = 0.030) in the expert goalkeepers.

## 1. Introduction

Decision making, cognitive (observational) processes, and attention focus are among the basic factors of soccer goalkeepers’ performance. Goalkeepers’ play is, in turn, part of the information processing mechanisms involved in various activities. First and foremost, it is important for goalkeepers to effectively perceive situations on the pitch and modify their defensive actions accordingly. The stages of information processing involve the identification/assessment of the opponent’s position and the appropriate choice of sensorimotor responses. Each originally formed response in the motor areas of the cerebral cortex is programmed in terms of control and regulation in the central nervous system. The control of the nervous system allows the development of a psychomotor response model, which is closely linked to perception mechanisms and decision-making processes. With the final response, a soccer player usually receives extrinsic biofeedback (environment and task timing) or intrinsic biofeedback (error or correct performance and muscle work) [1,2,3,4].

The model of sensory-motor response formation represents the foundation of Richard A. Schmidt’s concept of motor control [5,6]. The core of this model is the description of information processes, including the range of perceptual mechanisms in free motor activities. This concept of the control of human motor activities was extended by Zbigniew Czajkowski [7] by foregrounding the psychomotor response with a preparatory period (the fore period). According to Czajkowski, the effectiveness of athletes’ actions results from the advance of mental processes such as focus, concentration, and divided attention, and in the final action of phase-optimal decision making. This mechanism allows, then, the activation of a correct motor program (stored in motor memory) in anticipation of the expected stimulus. EMG, on the other hand, makes it possible to record the bioelectric tension of muscle activity, conditioning the proper execution of the action that affects the economy of effort and neuromuscular coordination.

To achieve a high level of efficiency, soccer players are required to accurately observe the situation on the field and coordinate muscle work (EMG muscle activity), which results in the optimal economy of effort. Perception is particularly important during difficult tactical actions, in which a soccer player should perform a task in accordance with an accepted movement pattern. Focused attention and effective perception increase movement economy, contributing to the reduction in so-called “noise” in the motor system (motor memory), which hampers fine movement control and makes the outcome of the movement less reliable [8]. Movement economy, in the case of EMG, most often refers to the shortening of reaction time; the shortening of “movement time”, which is the timing of the execution of the entire movement sequence [9]; and a reduction in muscular activity (muscle bioelectric tension) or the amount of motor units involved in a given range of motion [10]. When comparing expert athletes with novices, it can be assumed that a shorter motor action time indicates better neuromuscular coordination as well as a better focus of attention in making a dynamic final decision. Thanks to EMG, information can be obtained on neuromuscular responses in terms of the neurophysiological mechanisms of information processing during various motor activities.

While there are many strength and conditioning (S&C) training recommendations for outfield players, few publications can be found on soccer-specific S&C training in typical game situations for goalkeepers. This is similar in the case of mental training directed mainly at focusing attention. Goalkeepers fulfill an essential role in soccer and require special training; hence, the requirements for technical–tactical skills or the specifics of the defensive play of goalkeepers are markedly different from those of outfield players. There have also been no studies on soccer goalkeepers that used surface electromyography (EMG) as the main research tool. A few publications are suggested below that have been found to be helpful in delving into the subject of goalkeeping and focusing attention in selected sports.

The research achievements to date most often concern defending penalty kicks. At the moment, there have been no publications in which the research problem was typical fragments of the game in two-on-one situations. However, according to van der Kamp et al. [11], the activities of goalkeepers should take into account not only time-based decision making (the shorter, the more effective) but also the spatial aspects of decision making. The idea is to make the right decision when choosing the correct side of the goal (by simply guessing or relying on knowledge of the kicker’s preferred side) [12,13]. Timely defensive decision making reflects the relationship between the goalkeeper (i.e., the time taken to run and/or jump, including blocking the ball) and the penalty taker (i.e., the time available to pounce and block the ball) [11,14]. Highly skilled goalkeepers perform more agile movements, which allows them to cover more space in a short time [15,16]. 

In a study of young goalkeepers, Muracki [17] examined the impact of muscle soreness in different muscle groups on the performance of 5 m and 10 m speed tests with motor reaction choice (motion direction changes), i.e., activities based on real game situations. It turned out that the applied 5-day training cycle did not affect the shortening of the performance time of the motor speed test concerning motor reaction choice. The goalkeepers most often reported muscle soreness in their thigh muscles (the anterior compartment of the thigh, in particular) during the 5-day cycle, which confirms the greatest involvement of this muscle group in soccer goalkeepers’ performance.

Li et al. [18] attempted to determine expert and novice soccer goalkeepers’ perception of changes in open play situations with time constraints (limited display one-shot change detection paradigm) and without time constraints (continual cycling flicker paradigm). Unlike the novices, expert goalkeepers localized scene changes more accurately under time constraints and identified the changes more quickly when given sufficient time. The expert goalkeepers’ greater efficiency was likely due to pre-attentive processing; i.e., with sufficient time, they were able to focus on extracting detailed information for identification.

The goalkeepers’ effective action requires the finest physical and psychological states, for which understanding the mechanisms and factors is still under consideration. Previous papers have analyzed goalkeepers’ skills and abilities, especially during penalty kicks. To the best of our knowledge, no works on two-on-one game situations or on comparisons between seniors and youth have been published. Thus, to broaden the knowledge about goalkeeper-related actions, the main aim of the present study was to compare the decision-making time of soccer goalkeepers following a shot on goal and to determine the movement pattern structure (EMG) in goalkeepers in a typical game situation (two-on-one) in the context of play experience (seniors vs. youths).

The main research tool was surface electromyography (sEMG), and the following research hypotheses were adopted:

- Senior goalkeepers (Group A) will achieve a significantly shorter decision-making time during saving actions after the kicker’s shot on goal than youth goalkeepers (Group B);
- The movement pattern structure (EMG) will affect the economy of effort and eye–muscle coordination from the moment the attacker takes the shot to the goalkeeper’s completion of the save;
- Experienced senior goalkeepers display significantly lower muscle bioelectrical tensions compared to youth goalkeepers.

The rest of the paper is organized as follows: Section 2. Materials and Methods, which describes the subject characteristics and the study procedures and methods; Section 3. Results; Section 4. Discussion; Section 5. Conclusions; finally, a list of the references.

## 2. Materials and Methods

### 2.1. Subjects

The study involved 60 goalkeepers from Polish provincial teams, which were divided into two groups: seniors (*n* = 30) and youths (*n* = 30). Table 1 presents the basic data of goalkeepers from the senior group (A) and the youth group (B).

### 2.2. Procedures

The study had received approval from the Bioethics Committee of the Medical Chamber (Resolution 346 on 23 June 2022) in accordance with the Declaration of Helsinki guidelines for conducting clinical trials on humans using innovative research methods and tools.

The trials were performed using a 16-channel EMG system (Noraxon, DTS, Desktop Direct Transmission System, Scottsdale, AZ, USA) with a sampling frequency of 16 bits at 1500 Hz. Dedicated software was applied for data analysis (MyoResearch XP Master Edition for DTS Noraxon, Version 1.08.17). A wireless transmitter-recorder was used to synchronize the EMG system and transfer the EMG signal directly to the PC (3-axis wireless DTS 3D accelerometer sensor with ±6 g nominal output range, ±0.67 V/g sensitivity, and 5 Hz–1.8 kHz bandwidth).

The research was carried out in compliance with the principles of the SENIAM project [19]. The best trial out of three in a series of a given action from the right and left sides of the pitch was used for analysis (usually the second trial on each side of the pitch).

### 2.3. Methods

**Figure 1:**
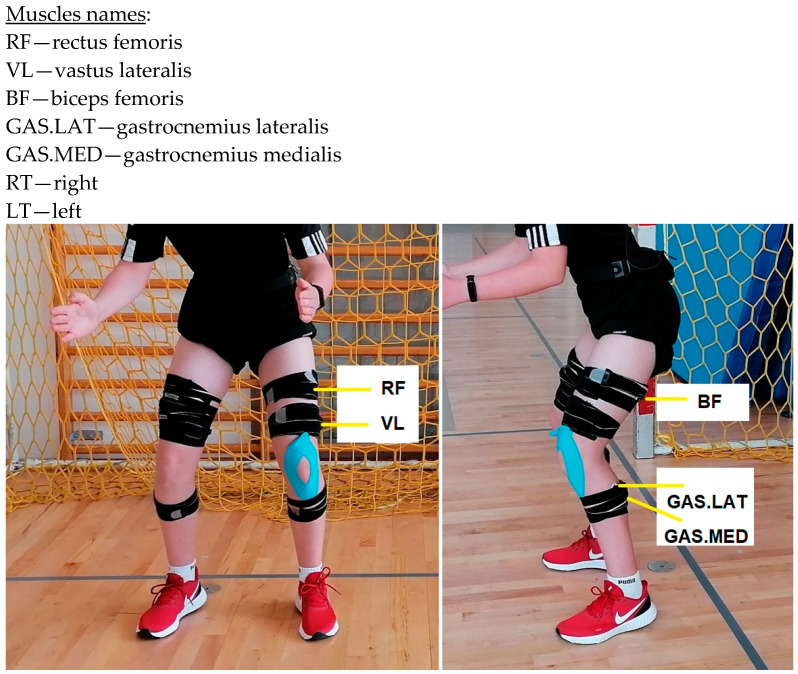
Placement of surface electrodes (anterior compartment of thigh muscles (RF RT and LT; VL RT and LT), posterior compartment of thigh muscles (BF RT and LT), and the gastrocnemius muscle (GAS.MED. RT and LT; GAS.LAT. RT and LT).

The trials were preceded by a 20–25 min individual warm-up with the trainer. Next, each goalkeeper had the electrodes placed on ten muscles: anterior compartment of thigh muscles (RF RT and LT; VL RT and LT), posterior compartment of thigh muscles (BF RT and LT), and the gastrocnemius muscle (GAS.MED. RT and LT; GAS.LAT. RT and LT) (Figure 1).

The adult goalkeepers (A) and parents of youth goalkeepers (B) filled out informed consent forms to participate in the study as well as personal image release forms. The players were provided with written instructions explaining all research procedures, and personal interviews were conducted.

**Figure 2:**
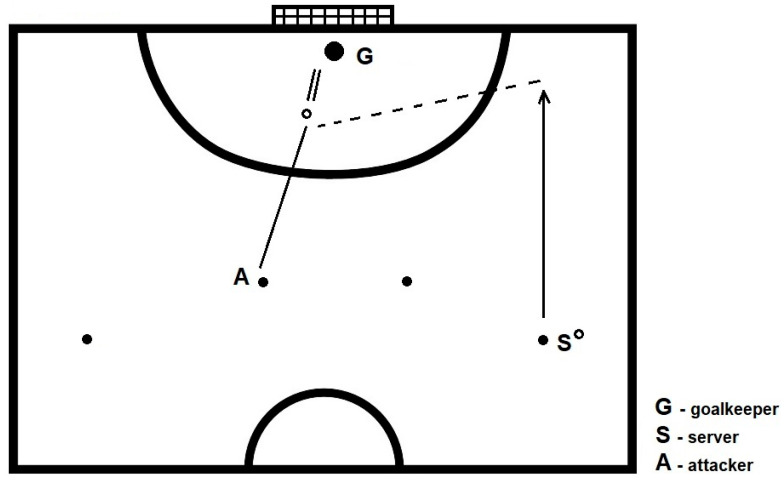
Positioning of players during a typical game situation (two-on-one): serving from the right side and then from the left side. The dotted line announce the passing of the ball and the arrows indicate the direction of the ball’s flight.

The positioning of players on the pitch and the game situation from passing the ball by the server (S) to receiving and striking the ball into the goal by the attacker (A) are shown in Figure 2. A series of three trials from each pitch side was carried out. Figure 2 demonstrates the game situation from the right side, and the same action (S–A) was performed three times from the left side of the pitch. The best attempt out of three from each side was taken for video analysis of shot-on-goal accuracy.

### 2.4. Statistical Analysis

The collected data were processed using StatsCloud (version 0.9.2). The study hypotheses were verified at the level of significance of *p* ≤ 0.05 (*t*-test and Mann–Whitney *U* test). The normal distribution of analyzed statistical data was checked with the Shapiro–Wilk test.

## 3. Results

**Figure 3:**
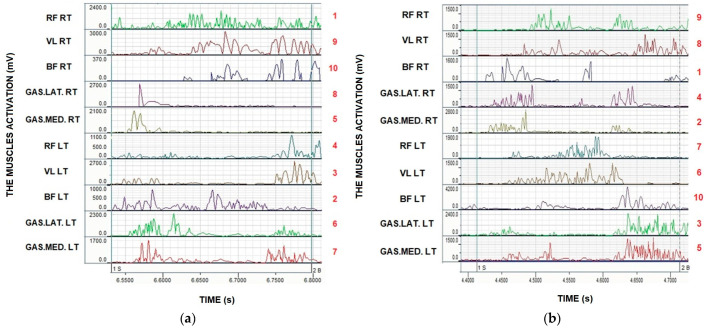
The pattern movement structure of selected leg muscles in senior goalkeepers (**a**) and youth goalkeepers (**b**).

The structure of muscle activation (the order in which muscles activate in a movement pattern) is presented in Figure 3a for the senior group and 3b for the youth goalkeepers. The muscle activation was analyzed from the moment of taking the shot by the attacker, throughout the pass (from the right and then the left side of the field) by the server, until the last phase of the goalkeeper’s saving action. The single best trial (out of three trials made from each side of the pitch) was used for analysis.

In the case of the senior goalkeepers (Group A), the game was played after a pass from the left side and the attacker struck the ball with his right foot, while in the youth goalkeeper group (Group B), the pass was made from the right side and the attacker struck the ball with his right foot.

After the shot, in the senior goalkeeper group (Group A, Figure 3a), the extensors of the right leg (RF RT) were activated first, followed by the flexors of the left leg (BF LT), and then the extensors of the left leg (VL LT and RF LT). Next, the gastrocnemius muscles (GAS.MED. RT and LT; GAS.LAT. RT and LT) were activated. At the final stage of the saving action, the right extensor (VL RT) was activated, and—considerably later—the right knee flexor (BF RT) was activated.

According to Figure 3b, in the youth goalkeeper group (Group B) the right knee flexors (BF RT) were activated first followed by the gastrocnemius muscles (GAS.MED RT and LT; GAS.LAT LT and RT) while simultaneously lifting off and shifting the center of gravity to the midfoot. At the end of the saving action, the muscles of the anterior compartment of the thigh, i.e., the knee extensors (VL LT and RT; RF LT and RT) and the flexor of the left leg (BF LT), were activated.

The identified movement pattern structure of the senior goalkeepers confirmed the hypothesis of the economy of effort and neuromuscular coordination of the postural muscles. The leading muscle groups, i.e., the quadriceps knee extensors (RF RT), are the first to activate, followed by the gastrocnemius muscles (GAS.MED. RT and LT; GAS.LAT. RT and LT). These muscle groups play a key role in goalkeepers’ jumping and diving for the ball.

**Chart 1:**
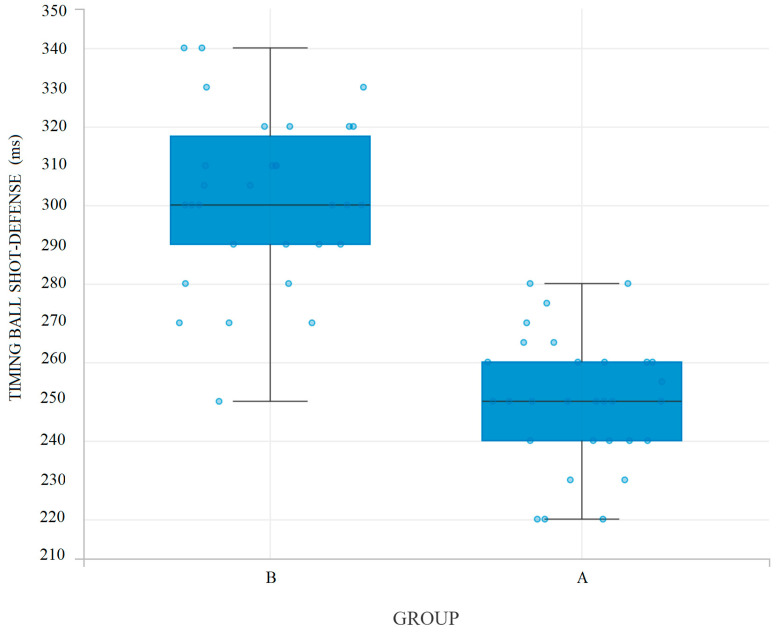
Differences in decision-making times by goalkeepers in a two-on-one game situation.

Another analyzed measure was the goalkeepers’ decision-making time (Groups A and B) in a typical game situation (two-on-one). Table 2 and Chart 1 indicate the significant differences in decision-making times in both groups of goalkeepers. The average time from the shot on goal to the goalkeeper’s completion of the saving action (the ball crossing the goalkeeper’s body range) in the senior group ranged from 250 to 260 ms, while in the youth group, it ranged from 300 to 320 ms. The youth group scored higher in saving action time than the senior group. The Mann–Whitney *U* test results showed the difference to be statistically significant (*p =* 0.001).

**Chart 2:**
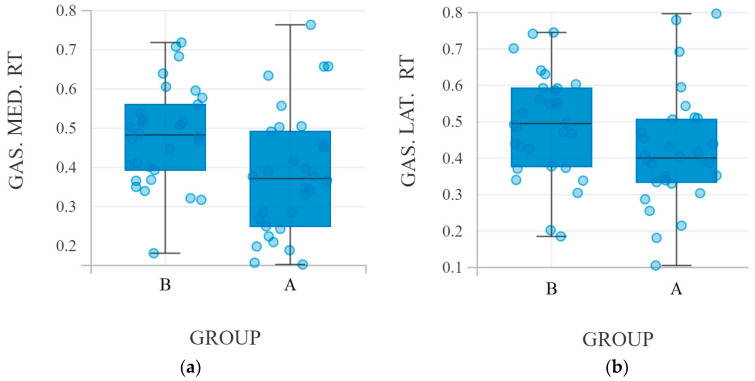
Differences between groups of goalkeepers (A and B) and % of maximum EMG signal values in the shot–defense phase. The charts (**a**,**b**) show the median % of maximal postural muscles EMG values among goalkeepers (GAS.MED and GAS.LAT).

The reliability of the obtained movement pattern structure is confirmed by the percentages of the maximum EMG signal values in the shot–defense phase shown in Table 3 and Chart 2a,b.

The obtained values clearly demonstrate significantly lower muscle bioelectric tensions (GAS.MED. RT—*p* = 0.008; GAS.LAT. RT—*p* = 0.030) in the senior goalkeepers compared to the youth goalkeepers. This confirms that some of the leading postural muscles in the movement pattern structure in goalkeepers are the calf muscles. This muscle group is what gives a goalkeeper the ability to jump for high balls.

## 4. Discussion

The aim of the present study was to compare goalkeepers’ decision-making times between seniors and youths in a typical two-on-one game situation in soccer. In addition, the movement pattern structure (EMG) and the bioelectrical tension of goalkeepers’ muscles were determined in a game situation from the attacker’s shot on goal to the final phase of the goalkeeper’s saving action.

Speed of perception, including developing so-called “reflexes”, is part of every goalkeeper’s training. It significantly affects a goalkeeper’s mobility and reaction speed during the game. In addition, the work of the muscular system is strongly coordinated with the work of the nervous system, in this case with decision-making speed. The timing of the decision making not only depends on when the goalkeeper decides to initiate a jump to the ball during the penalty taker’s run-up (e.g., whether to jump early or extend the reaction) but also on the players taking the shot (where and how to kick the ball (before the goalkeeper’s run/jump to one side of the goal ball or after). Therefore, making a decision relatively early (about 200 ms or longer before contact with the ball) during a shooting situation favors goalkeepers who force penalty kickers to change their strategy [16,20]. Upon this decision, the “reflex” of perceiving the correct direction in which to jump, also known as the psychomotor response, is developed. The goalkeeper’s posture in preparation for defense already results in an arrangement of muscle work that plays a key role in typical defensive situations. The goalkeeper’s stable posture requires neuromuscular control, including maintaining the optimal tone of postural muscles and the position of the center of gravity in the correct alignment. This gives goalkeepers the freedom of movement and locomotion in different directions and the ability to jump and dive for the ball [21,22,23,24]. High-class goalkeepers are characterized by more effective neuromuscular coordination. They are able to better manage the processes of perception (observation) and shape the stability of the position in various situations on the pitch. In addition, the research shows that better postural neuromuscular coordination and postural stability contribute to more efficient functional movements that are typical of individual sports [25,26,27]. Training programs that include general and sport-specific exercises that target the core and core muscles have been shown to improve body balance, strength, and the endurance of the back muscles [28,29]. Spinal stabilization and strengthening exercises improve postural and central stability and/or reduce back problems in athletes [30]. The core muscles provide the necessary stability for generating force in the lower limbs and the effective control of body movements [25].

The above analysis clearly confirms the research hypotheses. Firstly, the senior goalkeepers demonstrated significantly shorter decision-making time during their saving actions after the attacker’s shot on goal compared to the youth goalkeepers (Group A—250–260 ms; Group B—300–320 ms; *p* = 0.001). Our results are also confirmed by other authors who conducted research with the participation of goalkeepers.

The findings of Higueras-Herbada et al. [31] correspond to the results of the present study. The authors focused on the positioning of the goalkeeper’s hands during different directions of penalty kicks. Saves against low or high balls (vertical direction) were made about 245 ms after ball contact, while saves made against shots to the left and right side of the goal (horizontal direction) were made 115 ms before ball contact. Goalkeepers were also found to save penalties at middle heights more often than low and high penalties. Moreover, they predict the height of penalties less clearly than their horizontal distribution, more closely observing the kinematics of penalty takers. These results support the view that goalkeepers make the left–right decision by at least partly focusing on the kicker’s kinematics, and that they dynamically decide the vertical aspects of the movement later, focusing on the ball trajectory. According to many authors [32,33,34], however, training programs should focus more on the first part of the ball’s flight to improve the goalkeepers’ prediction of the height of penalty kicks. Ryu et al. [35] conducted a study to determine whether guided perceptual training supplemented with normal (unguided) training information would be effective in improving anticipatory skills. They found that a seven-day perceptual training cycle supplemented with guided information led to improvements in players’ perceptual anticipatory skills.

Secondly, the specific structure of the movement pattern contributed to effort economy and visual-muscular coordination. The economy of effort in this case testifies to the optimal range of motion of postural muscles in expert goalkeepers compared to novices. The quadriceps thigh extensors (RF RT) and calf muscles (GAS.MED RT and LT; GAS.LAT LT and RT) are crucial for the performance of soccer goalkeepers in situations of reaching and jumping for far balls. Neuromuscular coordination in the case of dynamic movements, such as jumping to the ball, requires full alertness and freedom of execution in order to avoid injury and trauma to muscles and ligaments. In addition, the economy of effort means that the body does not require more work, and this translates into a reduction in the motor units of the muscle groups involved in the movement. Thirdly, the senior goalkeepers demonstrated significantly lower bioelectrical tensions of GAS.MED RT (*p* = 0.008) and GAS.LAT. RT (*p* = 0.030) compared to youth goalkeepers. This confirms that some of the leading postural muscles in the movement pattern structure in goalkeepers are the calf muscles. This muscle group is what gives a goalkeeper the ability to jump for high balls. This type of fitness is greatly enhanced by plyometric training, which is extremely important in shaping not only the calf muscles (the gastrocnemius and the soleus) or the ankle plantar flexors (eccentric stretching followed by a dynamic take-off) but also in extending and flexing the Achilles tendon, which determines jumping performance. When reaching for the ball, proper strength training is also critical for goalkeepers’ quadriceps thigh muscle group (lowering the center of gravity and the moment of knee extension when jumping) and the aforementioned calf muscles. It is these two muscle groups that make it possible for a goalkeeper to be able to bounce on their toes.

Zachry et al. [8], in a study of basketball free throws, revealed that the throwers’ movement economy with reduced EMG muscle activity was enhanced by the effective coordination of agonist and antagonist muscle groups (the biceps and triceps of the shooting arm). In addition, it was found that the presumably increased “noise” in the motor system (i.e., increased EMG activity) resulting from internal focus (focus on activating a particular muscle in a given movement sequence) hindered precise movement control. The “noise” produced a less precise and reliable outcome. This is because players should focus more on the movement effect and not on the constituent parts of the movement. This determines the quality and efficiency of the movement pattern, which consequently increases the accuracy of movement execution. Reduced neuromuscular activity is, in turn, associated with an increase in movement accuracy. Reduced EMG activity (muscle bioelectrical tension) and, possibly, the decreased recruitment of motor units is indicative of improved movement accuracy and fluidity [10].

In real conditions, movement accuracy with the desired EMG activity contributes to greater movement speed, including a reduction in movement time and reaction time [8,36,37]. The optimal recruitment patterns of motor units can benefit tasks requiring maximal strength (e.g., high jump, discus throw, and even ball striking in soccer) and endurance (running, swimming, rowing, or cross-country skiing) [8]. In both cases, it would be appropriate to effectively engage muscle fibers to increase intramuscular and intermuscular coordination [38] so that only the appropriate (e.g., maximal) forces are generated at the right time and direction. Endurance activities, on the other hand, should benefit from the relatively low neuromuscular activity to generate an output power so that energy is conserved (working under conditions of economy of effort while resisting fatigue) or that a given level of activity is maintained over a longer period of time (in which the synchronization of motor units is a key factor in strength development).

There are also potential limitations associated with the present study. It should be noted that the subjects of our study were goalkeepers from Polish provincial teams. Players from higher-ranked leagues, including the top leagues, and international games, as well as players from the outfield, should be analyzed in future studies. Another limitation is that no psychological tests were conducted. As was mentioned above, the goalkeepers play their best matches not only when they are in the finest physical state but also when they are in the best mental state so that they may focus on the game. It seems to be important to plan analyses of psychological features as well as mental states in the next study design.

It is necessary to continue research to corroborate the findings of the present study. Goalkeepers’ decision-making time is a factor that is indispensable and that requires researchers’ constant attention to achieve better sports results. Automation in the area of neuromuscular coordination provides opportunities to effectively combine perception mechanisms with decision-making processes.

## 5. Conclusions

Our results suggest that senior goalkeepers are characterized by better parameters of motor control, muscle activation, and pattern movement (affecting the economy of physical effort) in comparison to youth goalkeepers. We postulate that the following findings may be useful in the planning of goalkeeper training processes:

1. The senior goalkeepers (Group A) (250–260 ms) achieved a significantly shorter decision-making time (*p* = 0.001) during saving actions after the kicker’s shot on goal than youth goalkeepers (Group B) (300–320 ms).
2. The movement pattern structure (EMG) affected the economy of effort and eye–muscle coordination from the moment the attacker took the shot to the goalkeeper’s completion of the save. The economy of effort testifies to the optimal range of motion of the postural muscles in expert goalkeepers compared to that of novices. The quadriceps thigh extensors (RF RT) and calf muscles (GAS.MED RT and LT; GAS.LAT LT and RT) are crucial for the performance of soccer goalkeepers in situations of reaching and jumping for far balls.
3. The experienced senior goalkeepers displayed significantly lower muscle bioelectrical tensions (GAS. MED. RT—*p* = 0.008; GAS. LAT. RT—*p* = 0.030) compared to youth goalkeepers.

## Figures and Tables

**Table 1:** Basic data of goalkeepers from the senior (expert) group, Group A, and the youth (novice) group, Group B.

Group		Age (Years)	Body Height (m)	Body Mass (kg)	Training Experience (Years)
A	Mean	22.00	1.89	88.63	12.88
	SD	2.35	0.07	10.99	3.85
B	Mean	15.38	1.83	71.38	8.75
	SD	1.32	0.09	11.27	2.28

**Table 2:** Statistical analysis (Mann–Whitney *U* test) of decision-making time from the shot on goal to the last phase of saving action in two groups of goalkeepers (senior and youth).

Group of Goalkeepers			Descriptive Statistics		Test Statistics		
Outcome	Predictor	Group	*n*	Median	U	z	*p*
Decision-making time	Group	Youths (B)	30	300	28.5	−6.254	<0.001 *
		Seniors (A)	30	250			

* *p* < 0.05

**Table 3:** Statistical analysis (Mann–Whitney *U* test) between goalkeeper groups (A and B) and % of maximum EMG signal values in the shot–defense phase.

Group of Goalkeepers			Descriptive Statistics		Test Statistics		
Outcome	Predictor	Group	*n*	Median	*U*	*z*	*p*
GAS.MED. LT	Group	B	30	0.444	437	−0.192	0.848
		A	30	0.451			
GAS.LAT. LT	Group	B	30	0.473	437	−0.192	0.848
		A	30	0.436			
BF LT	Group	B	30	0.479	418	−0.473	0.636
		A	30	0.518			
VLO LT	Group	B	30	0.543	422	−0.414	0.679
		A	30	0.541			
RF LT	Group	B	30	0.555	408	−0.621	0.535
		A	30	0.507			
GAS.MED. RT	Group	B	30	0.481	271	−2.646	0.008 *
		A	30	0.370			
GAS.LAT. RT	Group	B	30	0.493	303	−2.173	0.030 *
		A	30	0.399			
BF RT	Group	B	30	0.505	379	−1.050	0.294
		A	30	0.474			
VLO RT	Group	B	30	0.506	427	−0.340	0.734
		A	30	0.501			
RF RT	Group	B	30	0.478	379	−1.050	0.294
		A	30	0.536			

* *p* < 0.05

## Data Availability

The data used to support the findings of this study are included within the article.
